# Supplementary material for: Synthesis and Evaluation of the Antifungal and Toxicological Activity of Nitrofuran Derivatives
Source: Pharmaceutics. 2022 Mar 8;14(3):593. doi: 10.3390/pharmaceutics14030593 (PMC8950151; doi:10.3390/pharmaceutics14030593)
Supplement: Supplementary file 1 [file pharmaceutics-14-00593-s001.zip › pharmaceutics-1609905-supplementary.pdf]

Supplementary Materials: Synthesis and Evaluation of the Antifungal and Toxicological Activity of Nitrofuran Derivatives

Carolina Orlando Vaso, Fabiana Pandolfi, Níura Madalena Bila, Daniela De Vita, Martina Bortolami, Maria José Soares Mendes-Giannini, Valeria Tudino, Roberta Costi, Caroline Barcelos Costa-Orlandi, Ana Marisa Fusco-Almeida and Luigi Scipione

Table S1. list of CAS number of nitrofurans **4-14.**

Compound	Structure	CAS	Compound	Structure	CAS
**4**	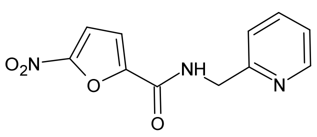	330466-62-1	**10**	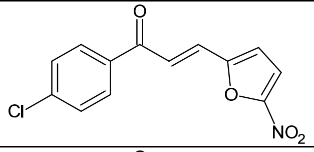	74470-48-7
**5**	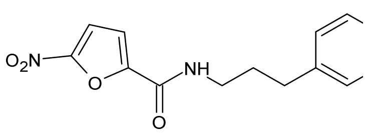	908539-64-0	**11**	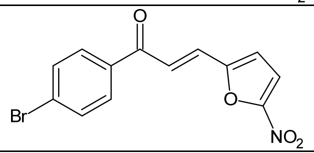	74470-50-1
**6**	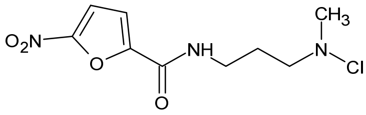	330468-41-2	**12**	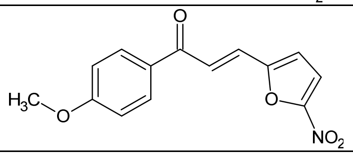	74470-56-7
**7**	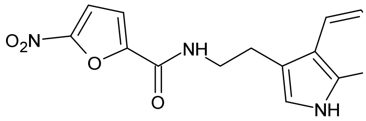	796056-49-0	**13**	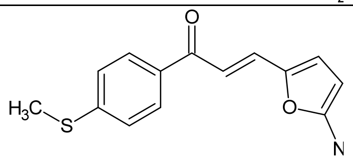	2101758-58-9
**8**	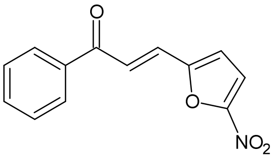	67001-70-1	**14**	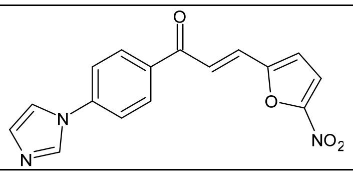	2101758-48-7
**9**	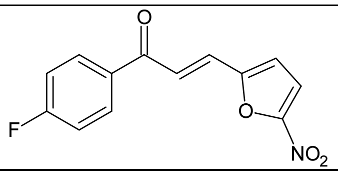	74470-51-2			

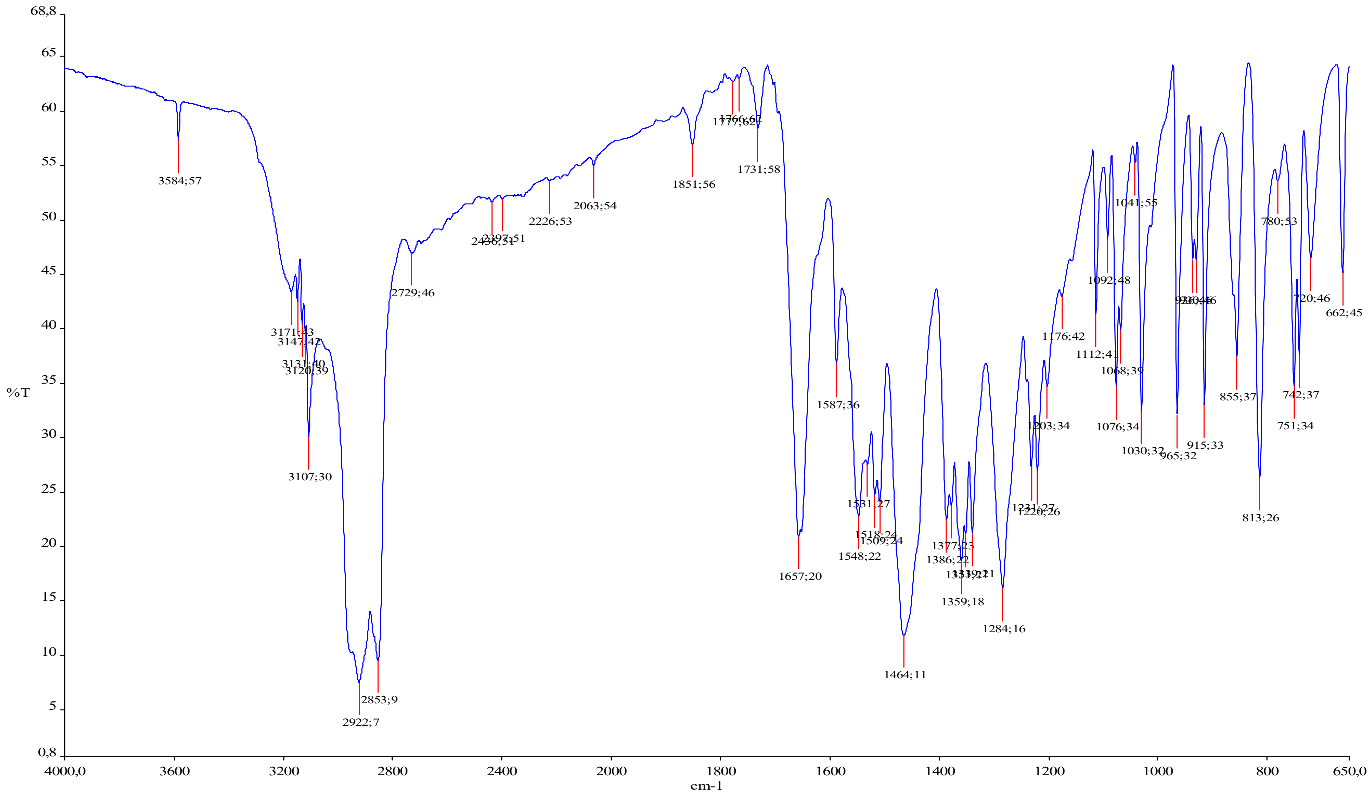

Figure S1. IR spectra of (nujol mull) compound **2**.

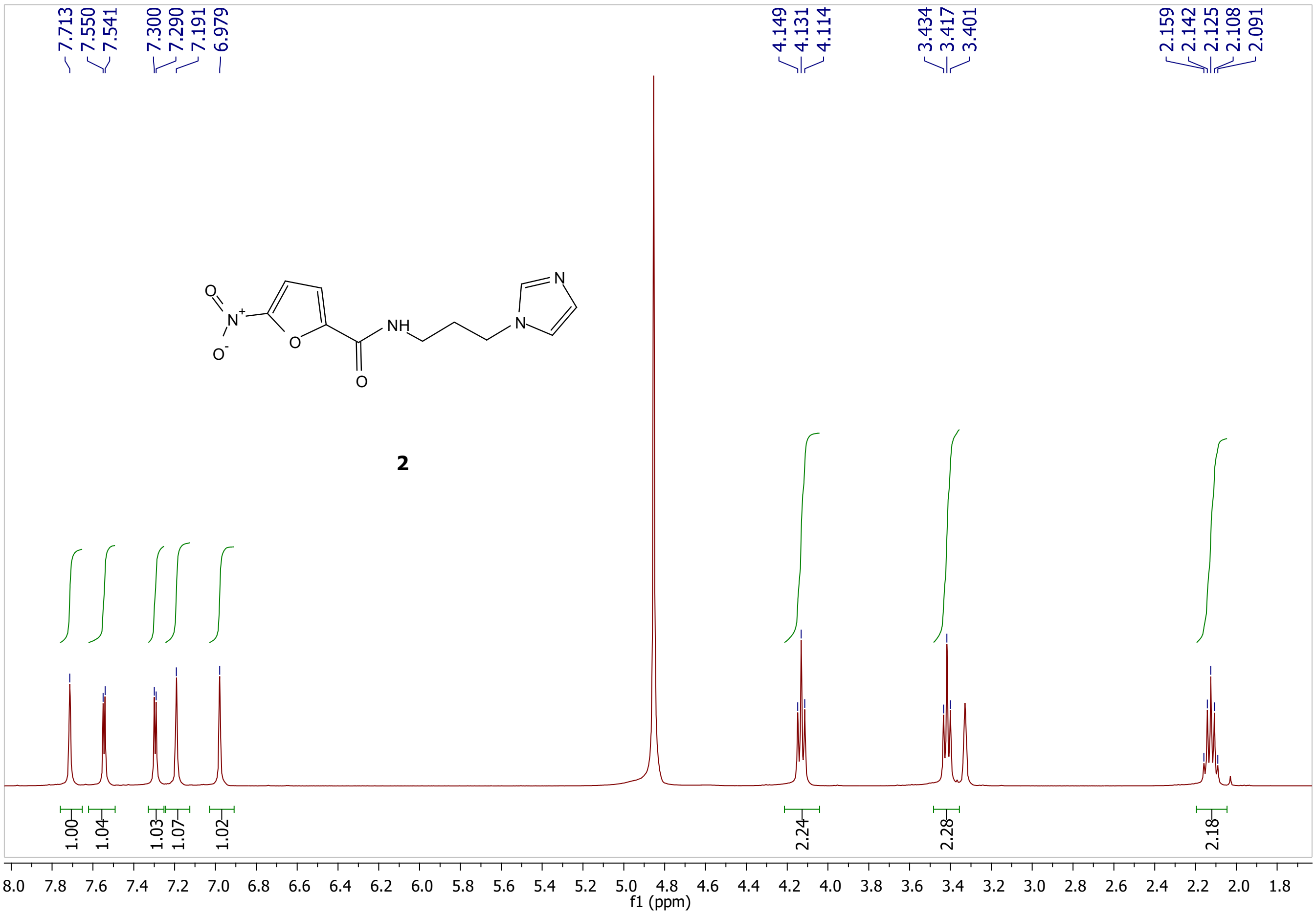

Figure S2.^{1}H NMR spectra of (400 MHz, MeOD) compound **2.**

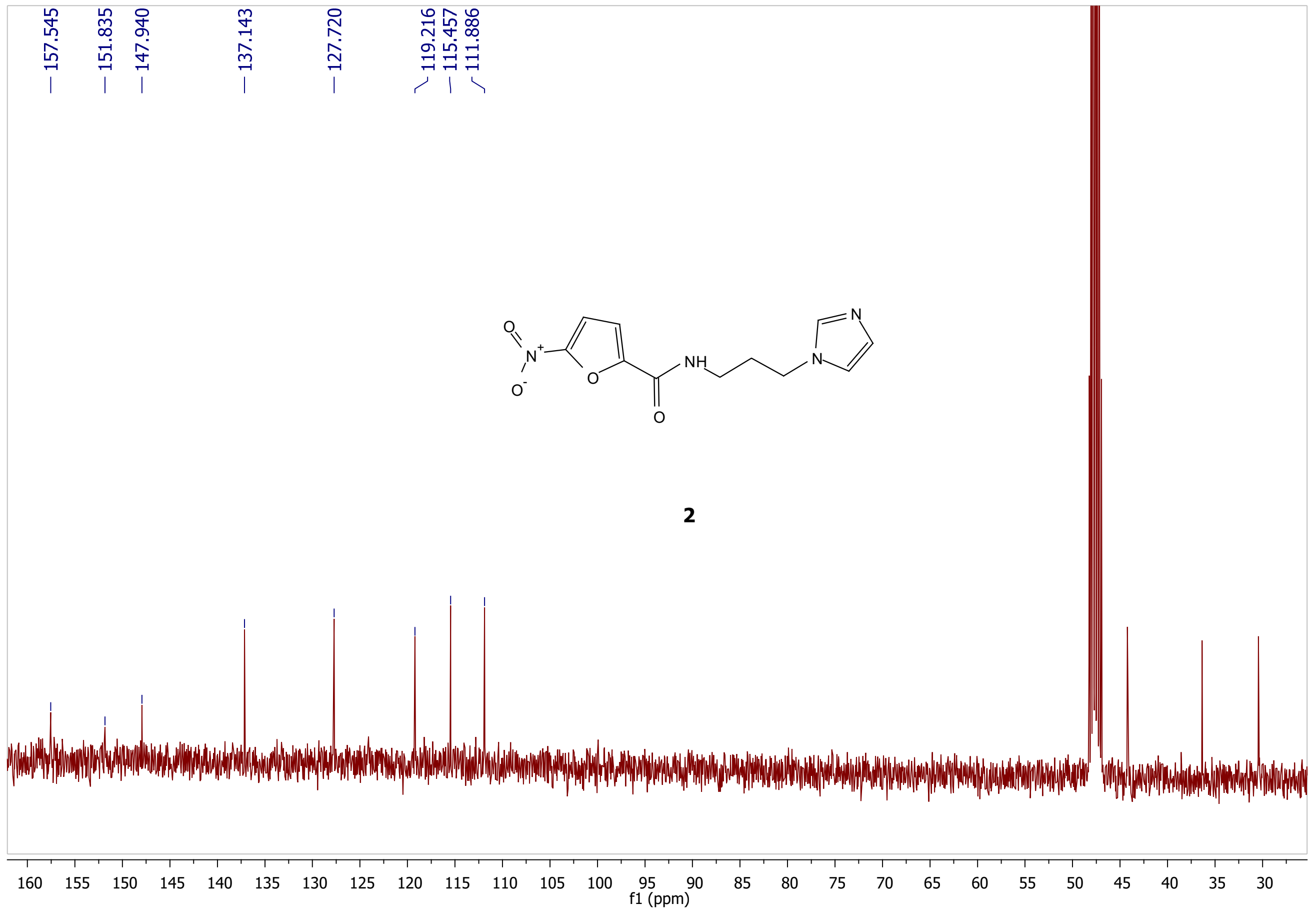

Figure S3. ^{13}C NMR spectra of (100 MHz, MeOD) compound **2**.

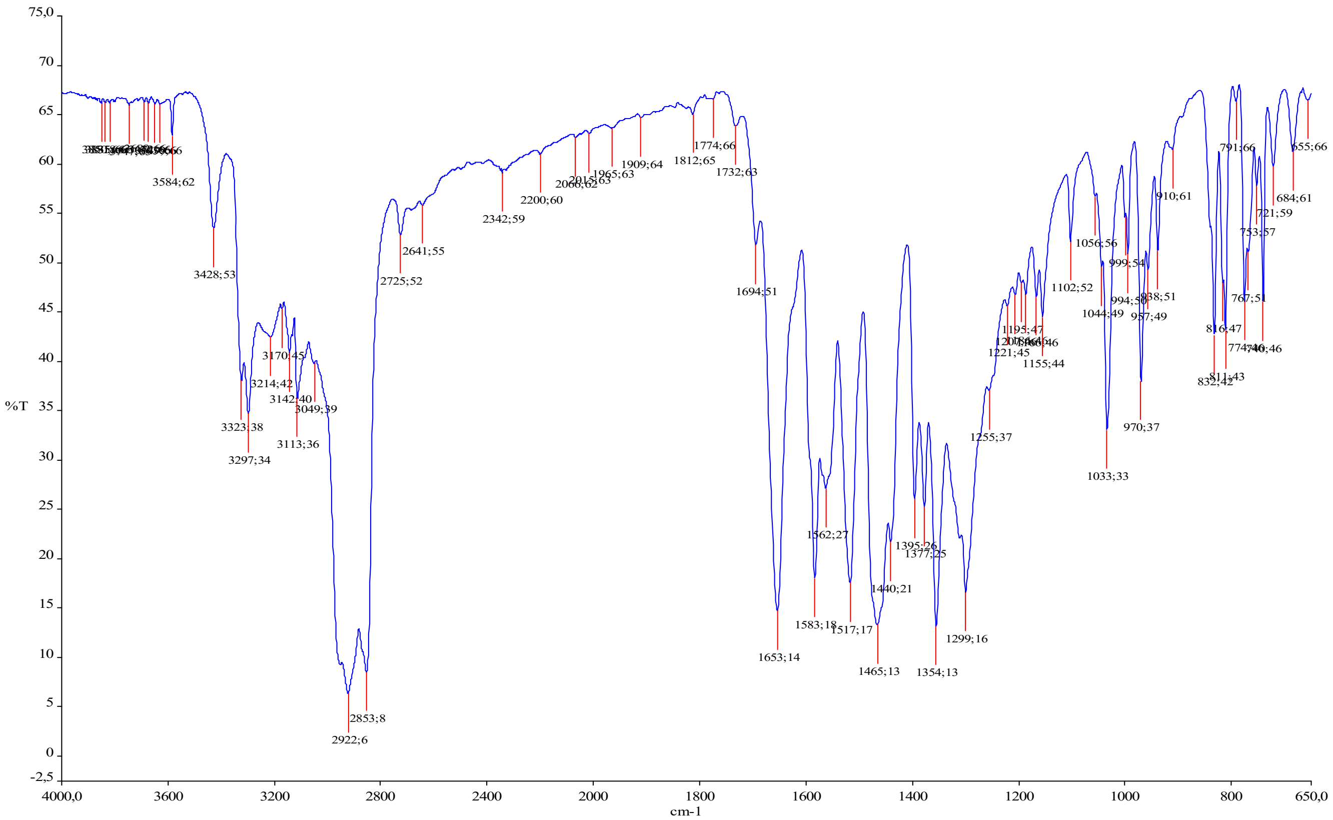

Figure S4. IR spectra of (nujol mull) compound **3**.

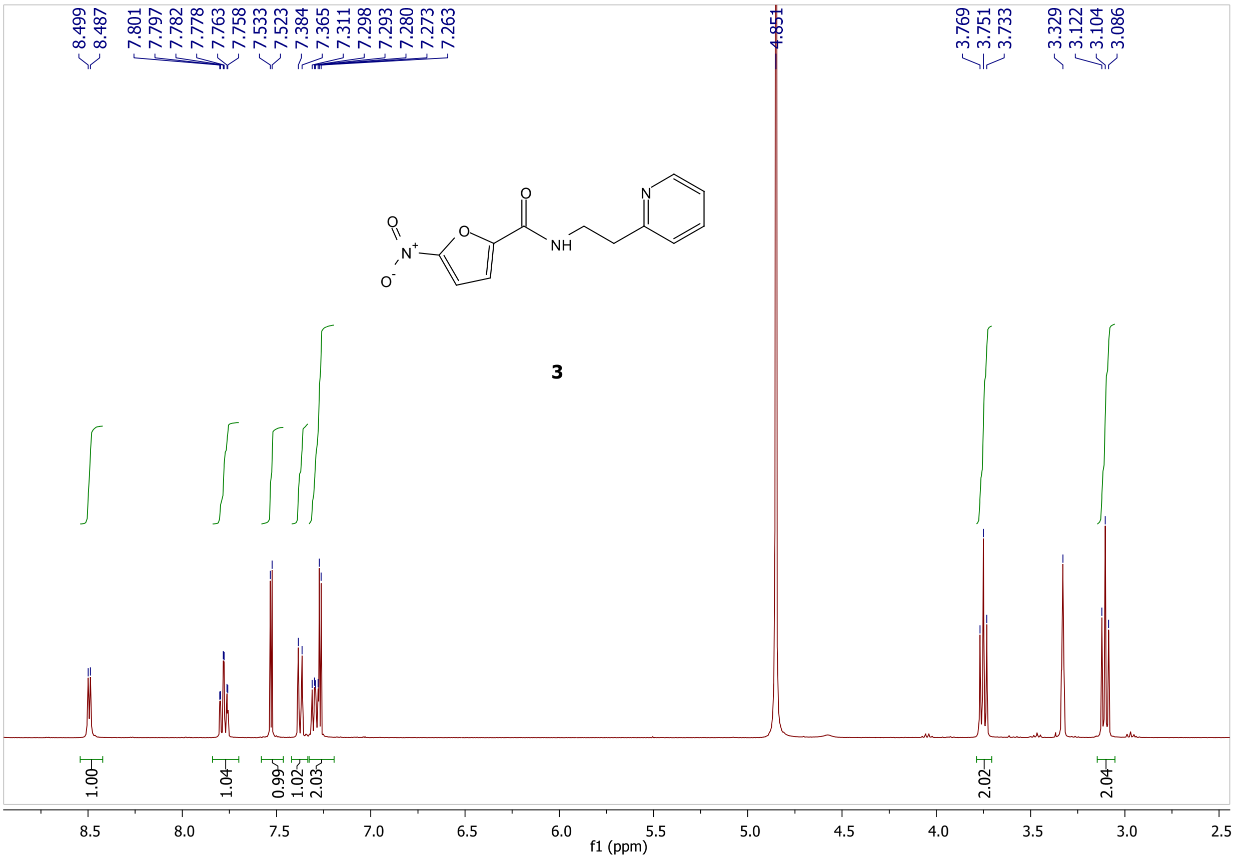

Figure S5.^{1}H NMR spectra of (400 MHz, MeOD) compound **3.**

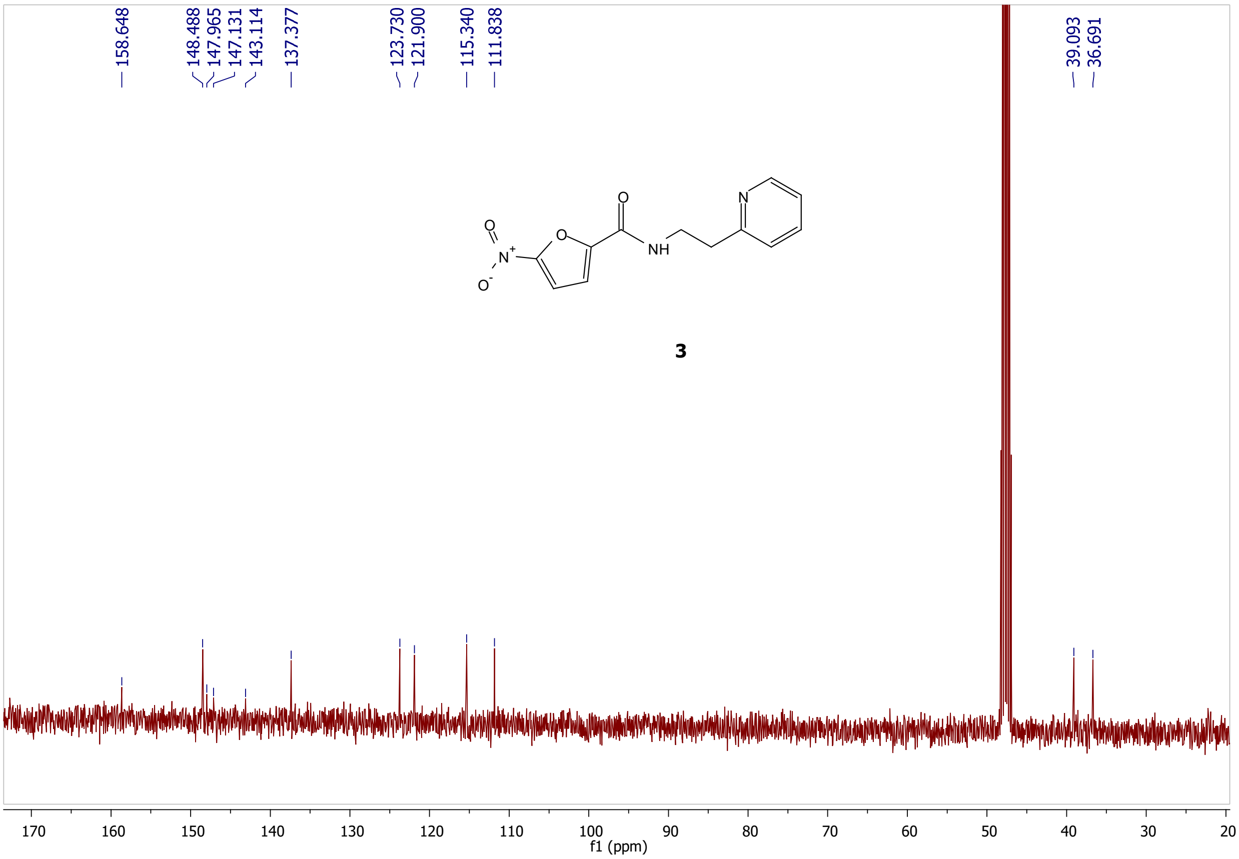

Figure S6. ^{13}C NMR spectra of (100 MHz, MeOD) compound **3**.

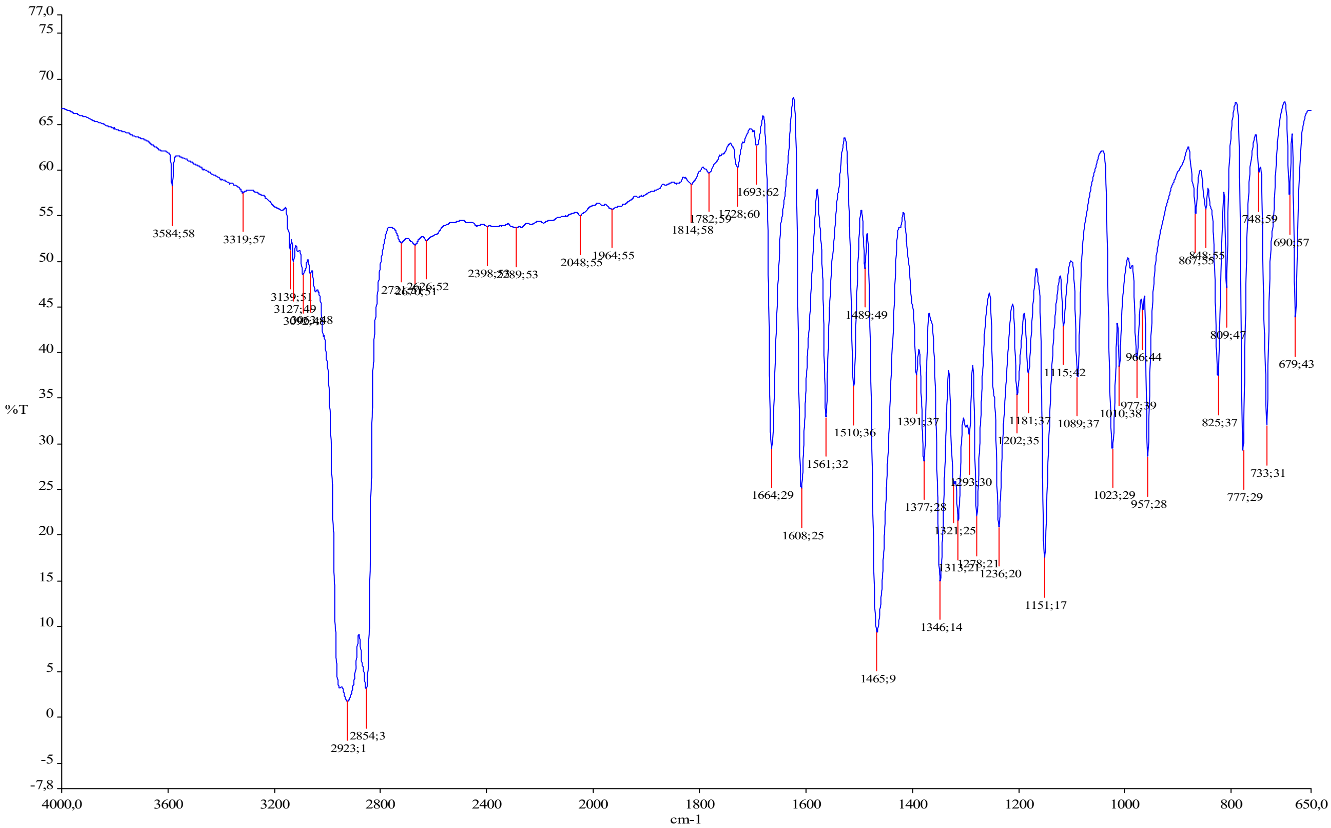

Figure S7. IR spectra of (nujol mull) compound **15.**

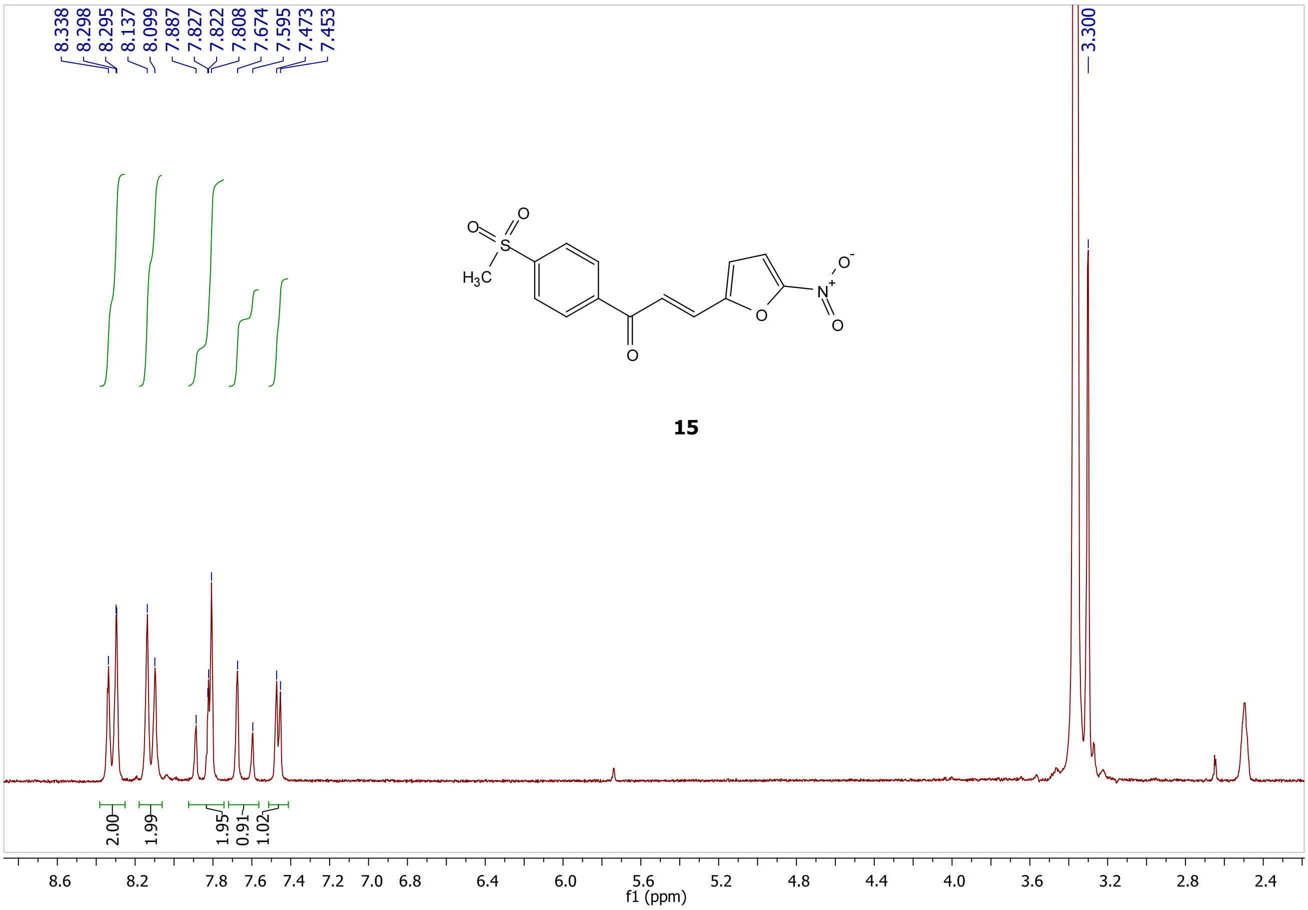

Figure S8.^{1}H NMR spectra of (400 MHz, DMSO-d_6) compound **15.**

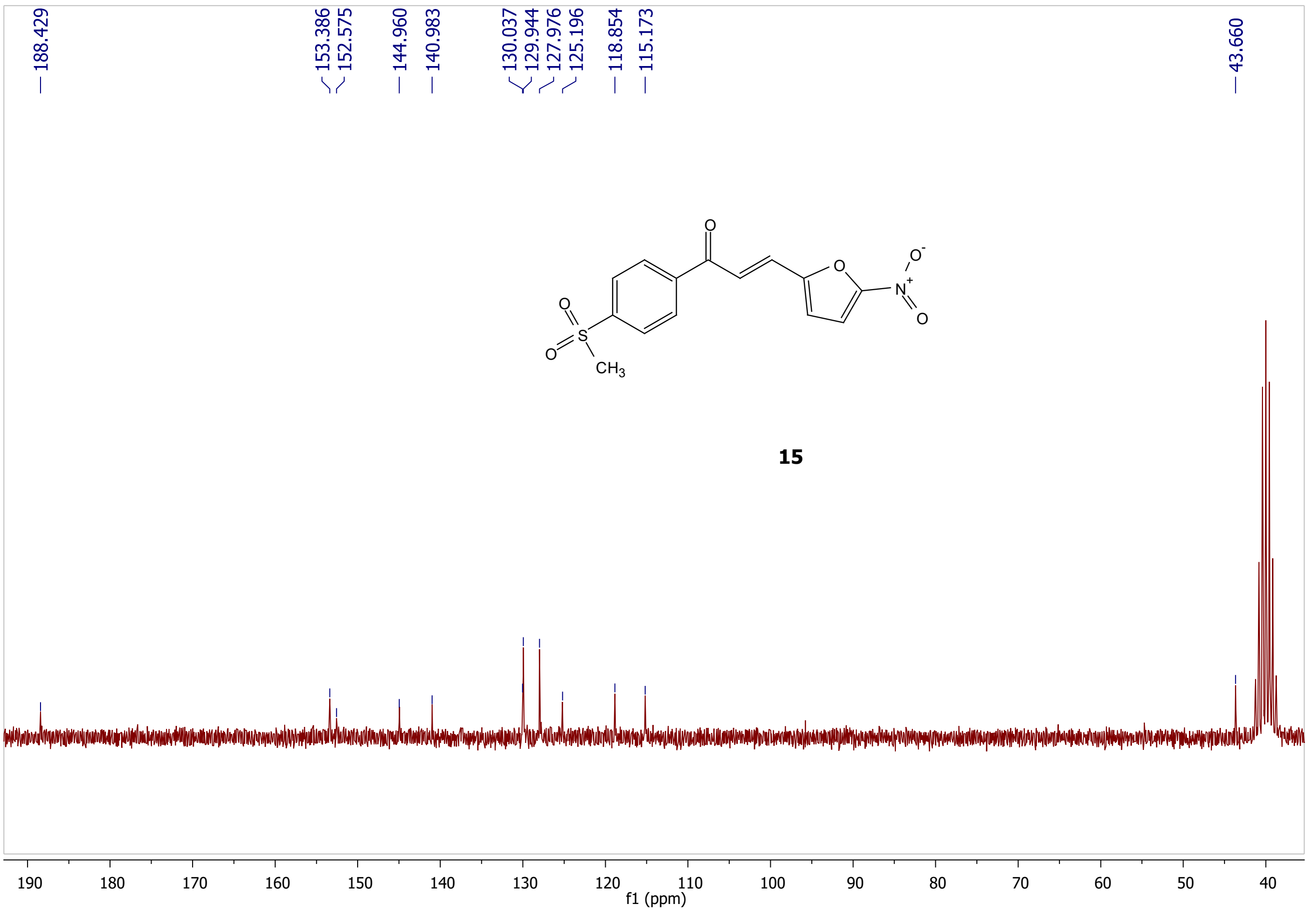

Figure S9. ^{13}C NMR spectra of (100 MHz, DMSO-d_6) compound **15**.

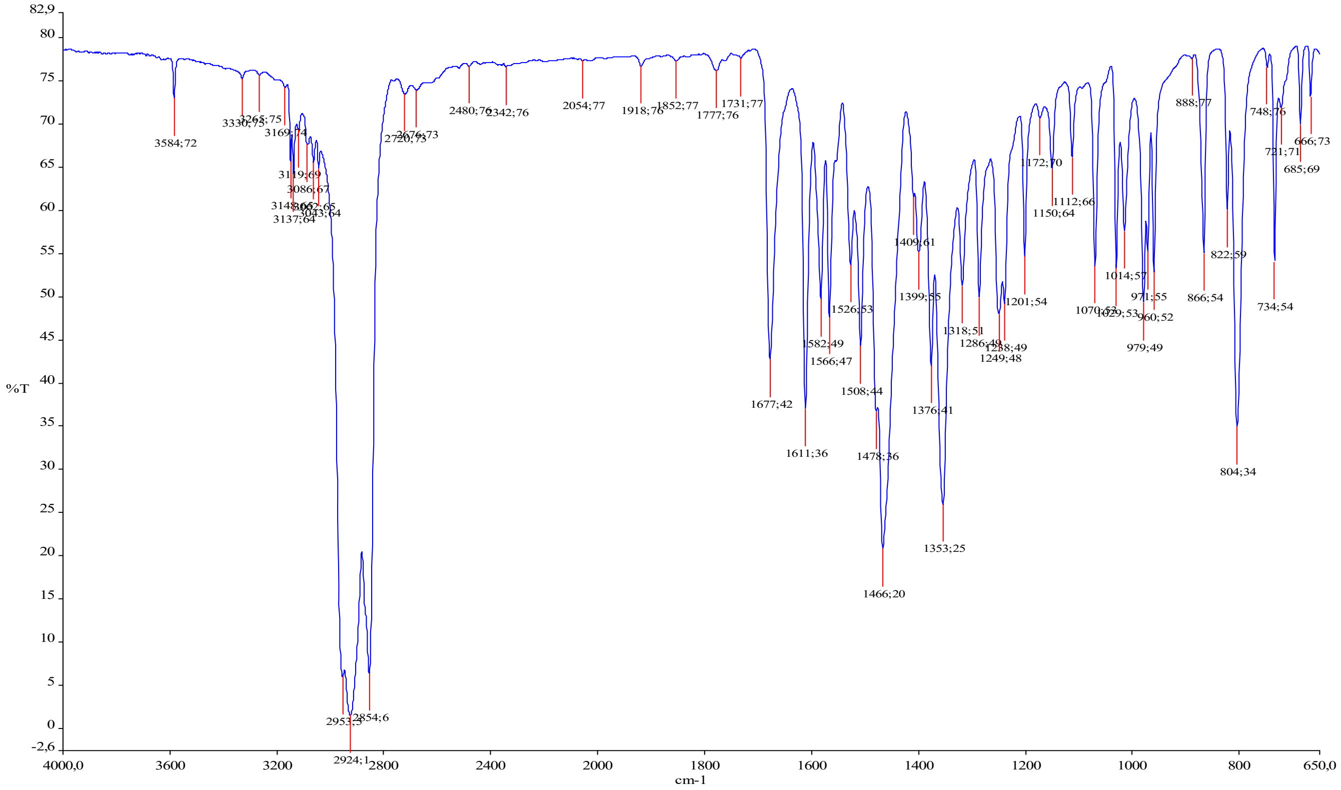

Figure S10. IR spectra of (nujol mull) compound **16.**

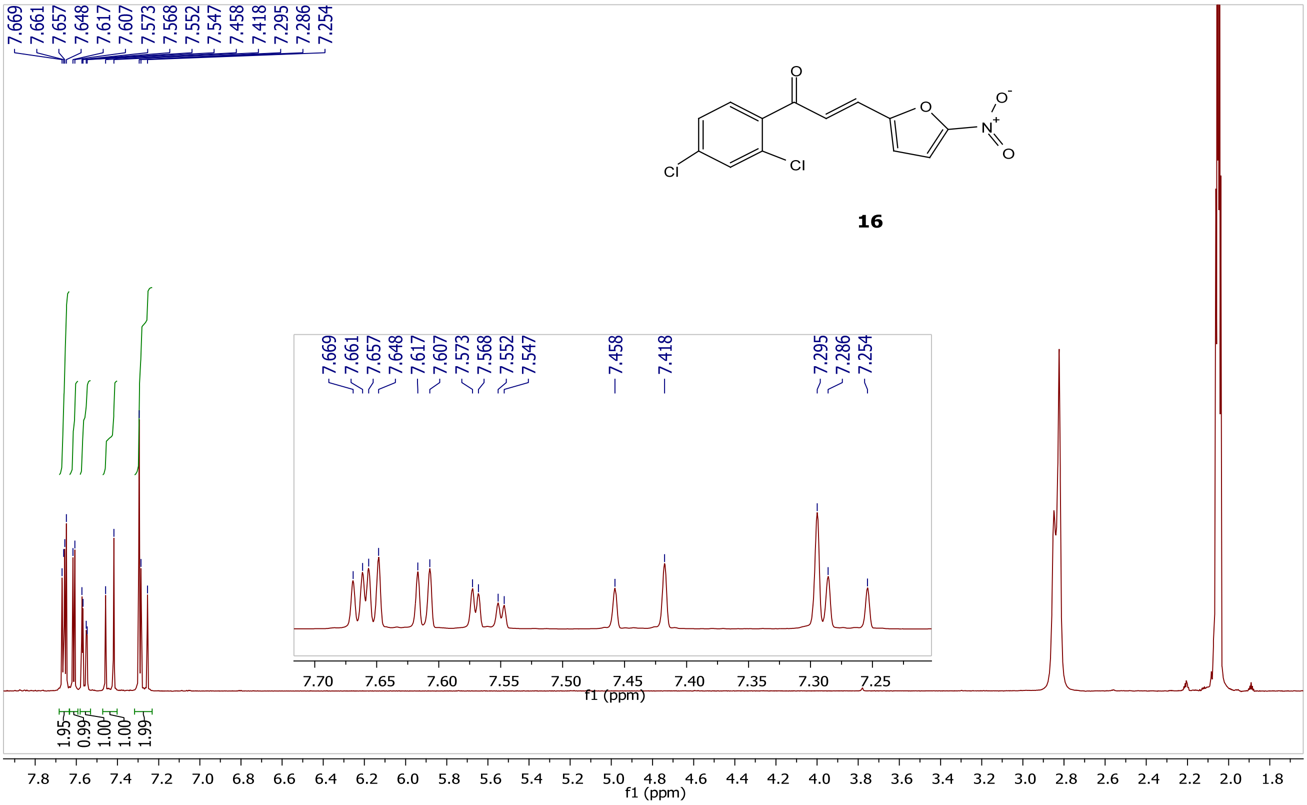

Figure S11.^{1}H NMR spectra of (400 MHz, Acetone-d_6) compound **16.**

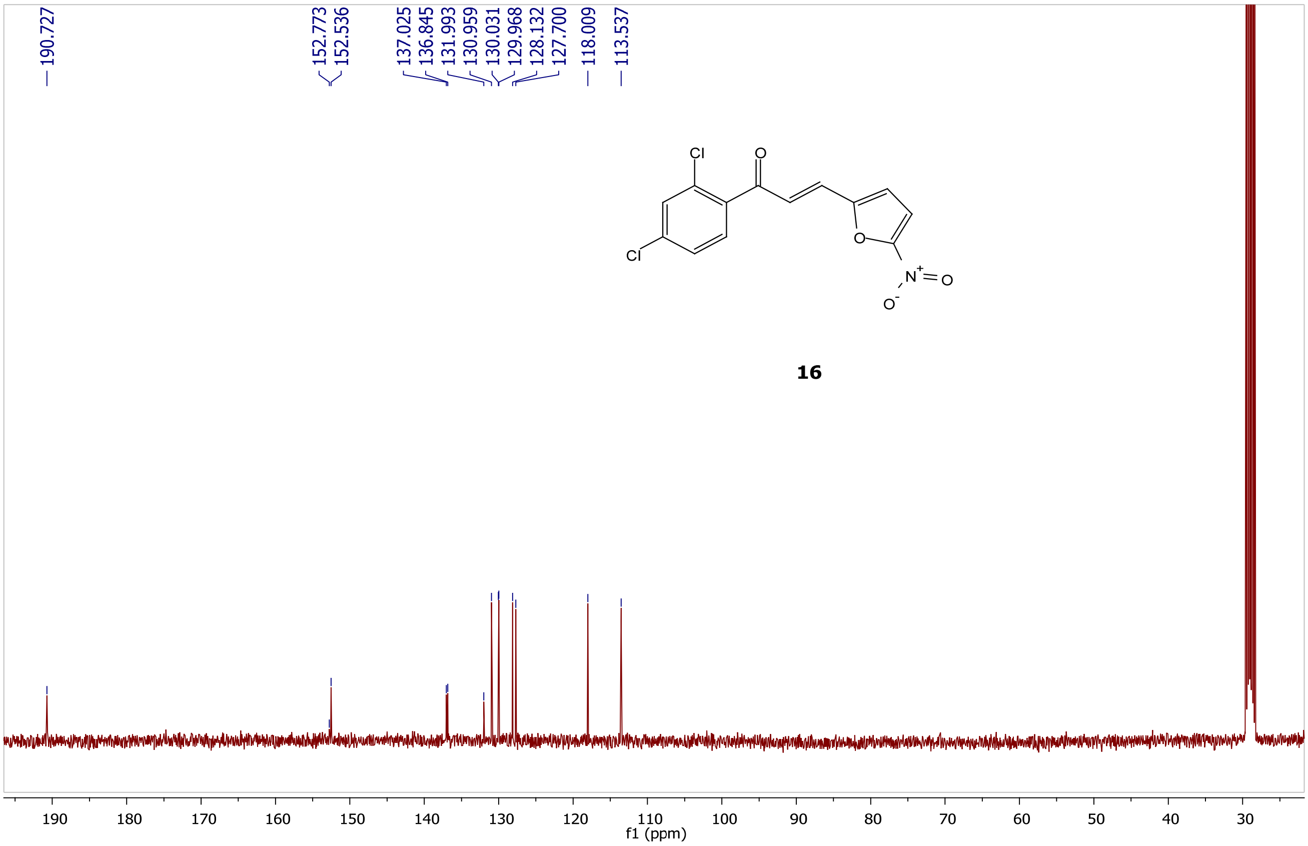

Figure S12. ^{13}C NMR spectra of (100 MHz, Acetone-d_6) compound **16**.

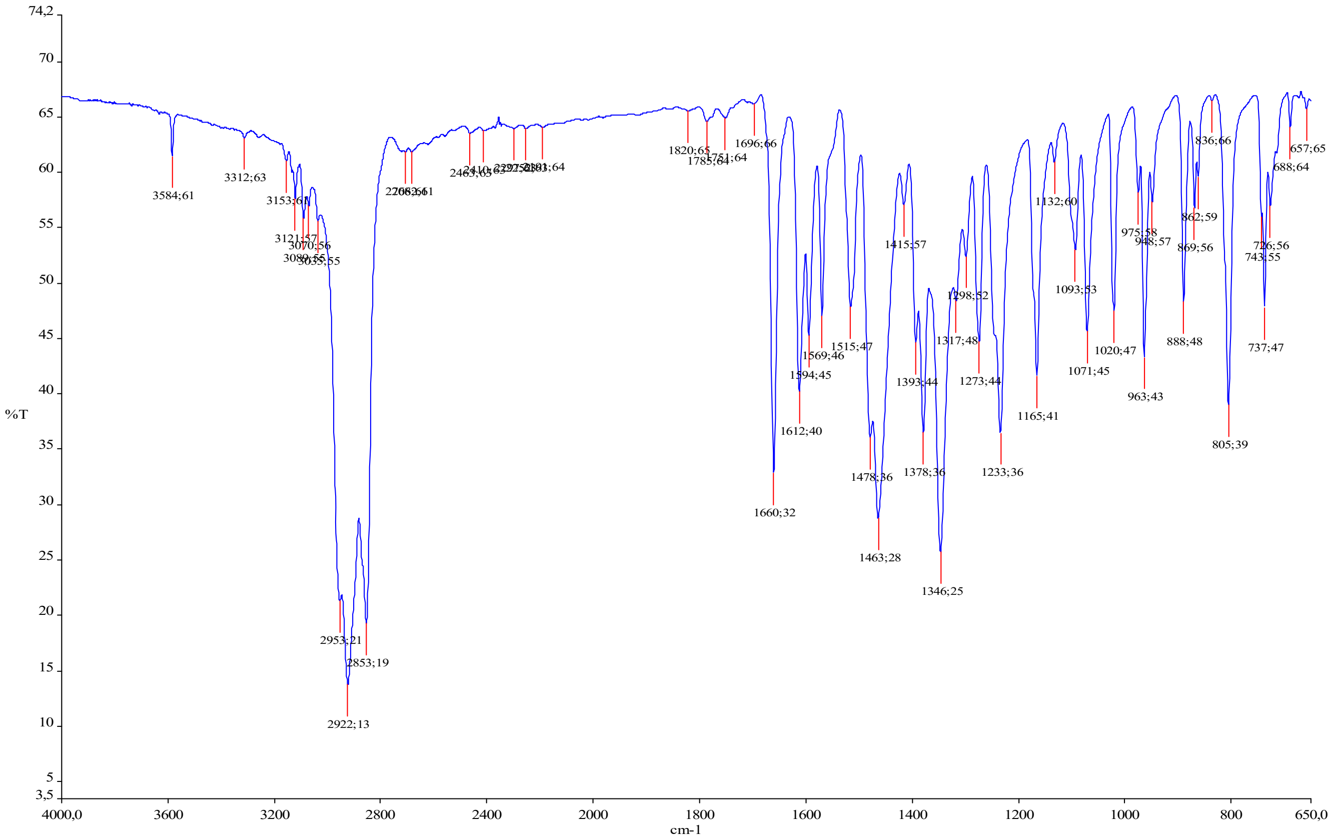

Figure S13. IR spectra of (nujol mull) compound **17.**

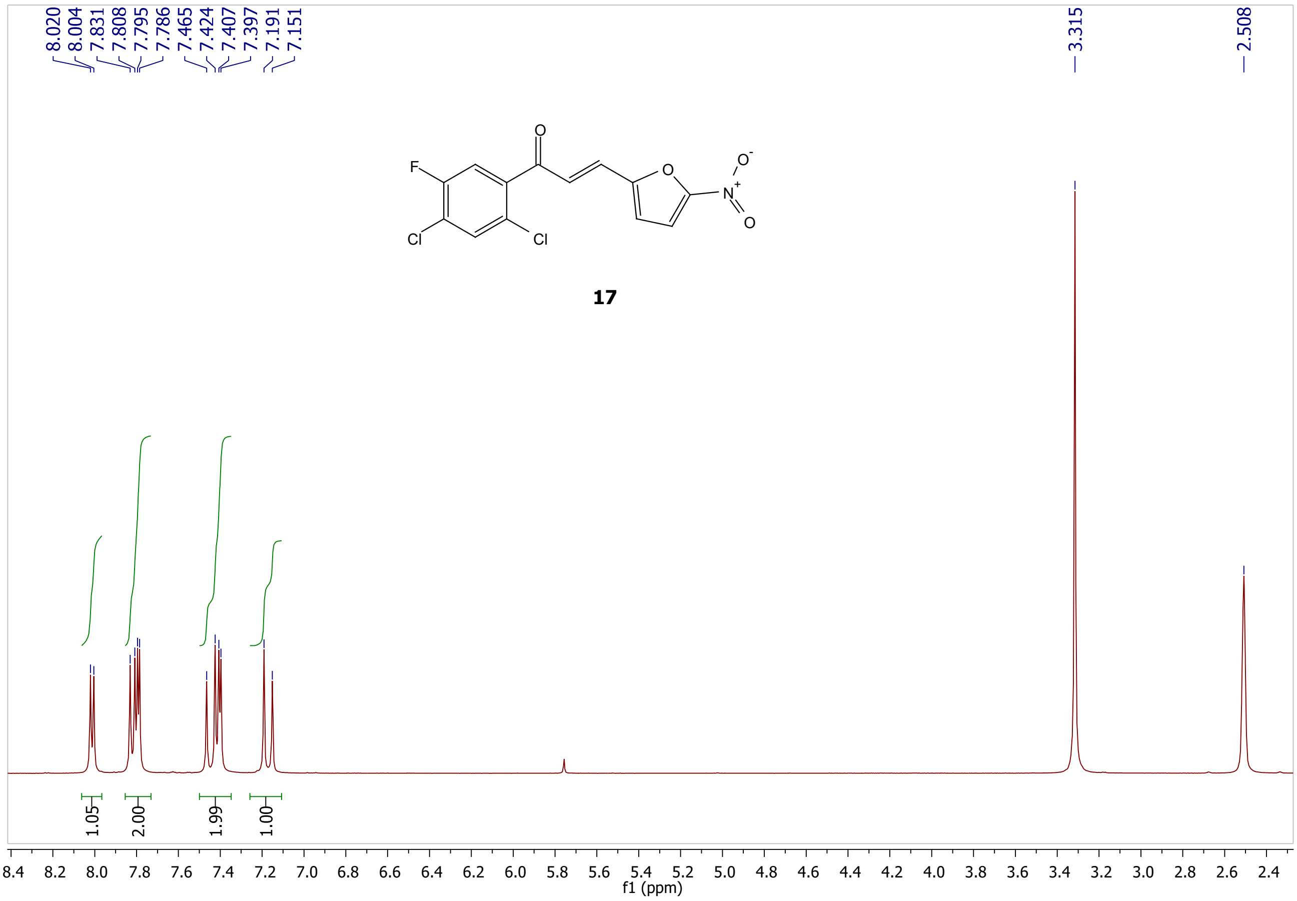

Figure S14.^{1}H NMR spectra of (400 MHz, DMSO-d_6) compound **17.**

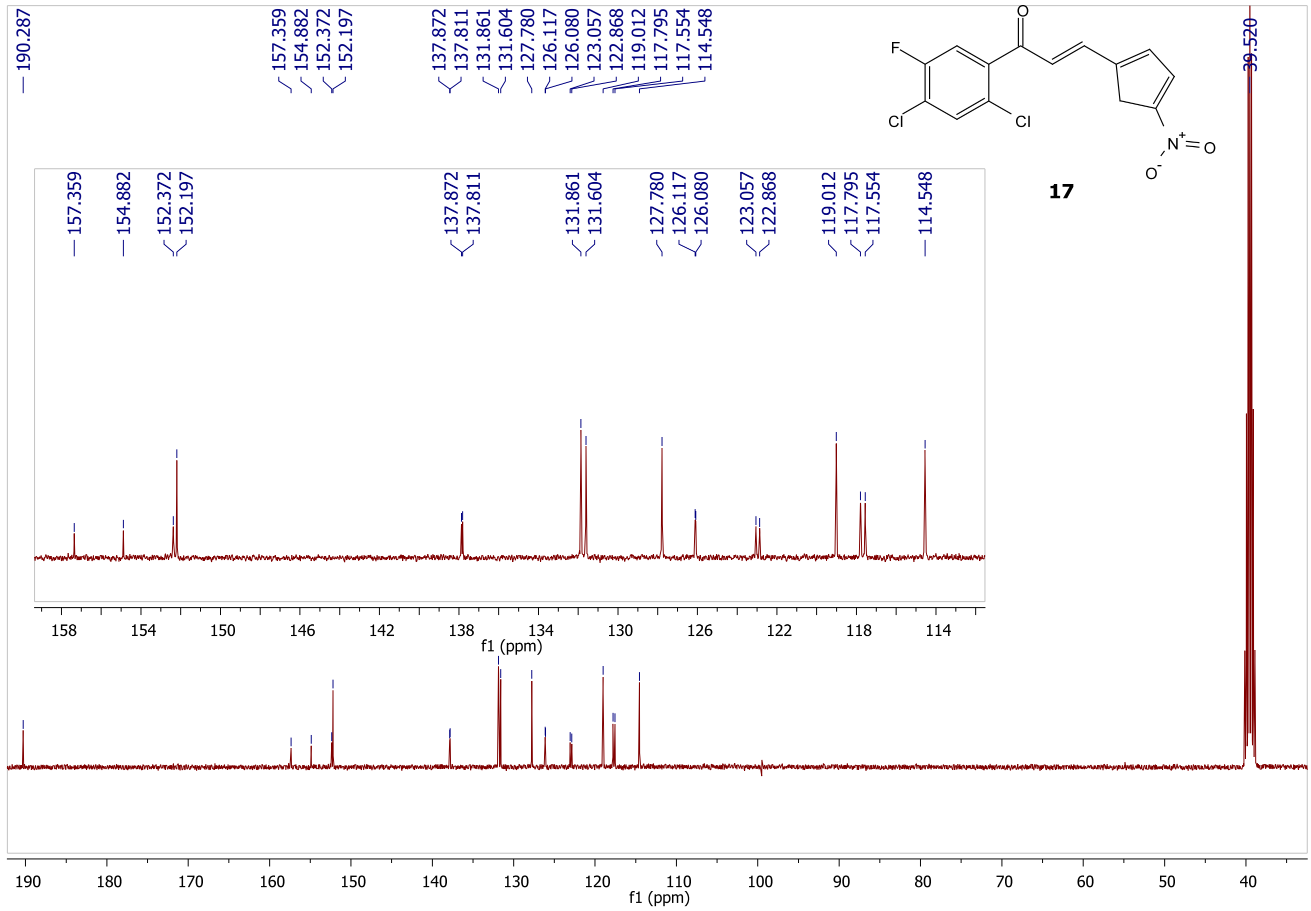

Figure S15. ^{13}C NMR spectra of (100 MHz, DMSO-d_6) compound **17.**
